# Don't wait any longer, conceive in time: a systematic review and meta-analysis based on semen parameters after varicocelectomy

**DOI:** 10.1007/s11255-024-04080-y

**Published:** 2024-05-18

**Authors:** Yangyang Mei, Nuo Ji, Xingliang Feng, Renfang Xu, Dong Xue

**Affiliations:** 1 Department of Urology, Jiangyin People’s Hospital of Jiangsu Province, Jiangyin, Jiangsu China; 2 Department of Urology, The Third Affiliated Hospital of Soochow University, Changzhou, Jiangsu China

**Keywords:** Varicocele, Varicocelectomy, Semen parameters, Meta-analysis, Systematic review

## Abstract

**Background:**

Varicocelectomy was considered to be beneficial to patients with varicocele-related infertility. However, there are only a few researchers who have explored the relationship between better timing and postoperative semen improvement in patients.

**Methods:**

We conducted this meta-analysis by enrolling published prospective studies to find out the best waiting time after varicocelectomy to wait for better improvement of semen quality. An extensive search was conducted in PubMed, Web of Science, and Cochrane Library to identify eligible studies. The included studies were then analyzed comprehensively using STATA software and standardized mean differences (SMDs) and their corresponding 95% confidence intervals were calculated.

**Results:**

Our comprehensive analysis showed that after varicocelectomy, follow-up results within 3 months or longer showed a significant improvement in semen parameters compared to the preoperative period. Notably, no further improvement in semen parameters was observed when the follow-up period reached six months or longer (semen volume: WMD: − 0.07 (− 0.29, 0.16); sperm concentration: WMD: − 1.33 (− 2.33, − 4.99); sperm motility: WMD: 2.31 (− 0.55, 5.18); sperm morphology: WMD: 1.29 (− 0.66, 3.24); sperm total motile count: WMD: 3.95 (− 6.28, 14.19)).

**Conclusions:**

Three months after varicocelectomy may be the optimal time for semen parameters compared to six months or even longer, which means it is also the preferable time for conception. However, more well-designed prospective studies are needed in the future to validate our conclusion.

## Introduction

Varicocele, defined as abnormal dilation and tortuosity of internal spermatic veins within the pampiniform plexus [1, 2], is regarded as the most common surgically correctable cause of infertility in men [3]. The prevalence of varicocele in the healthy adult male population was about 15%, in men with primary infertility the prevalence was approximately 35–44%, and the index increased to 45–81% in men with secondary infertility [4–6]. Therefore, it is widely recognized that varicocele can affect the quality of semen in men. Several mechanisms could explain the resultantly impaired semen quality of varicocele, as follows: (i) overproduction of reactive oxygen species [7]; (ii) decrease of antioxidant level [8]; (iii) increase of scrotal temperature [9]; (iv) sperm DNA damage [10].

It is internationally accepted that varicocelectomy is an effective treatment for varicocele and can be helpful for patients who suffer from varicocele combined with infertility [11]. Initially, patients with varicocele were more concerned about natural conception rates after surgical treatment [12]. Gradually, the positive effects of varicocelectomy on semen quality have attracted more and more attention from clinicians and infertility couples. In general, about 60–70% of men achieve sperm improvement after varicocelectomy16 and there have been several meta-analyses demonstrating this fact [13–16]. Among studies comparing the sperm parameters before and after varicocelectomy, the follow-up time after varicocelectomy varied a lot, ranging from 3 to 12 months [17–19]. However, the sample sizes of these studies were not sufficiently large, and there are no meta-analyses to compare improvements in semen parameters across follow-up times.

For patients and clinicians, it is essential to know the best time to wait for better improvement of semen quality after varicocele surgery. Initially, the recommended waiting time by the Practice Committee of the American Society for Reproductive Medicine was at least 12 months [20]. Later, they changed their recommended waiting time to 3–6 months after varicocele surgery, corresponding to 1–2 normal spermatogenic cycle [21]. The studies on different waiting times after varicocelectomy are well designed and conducted but enrolled less study population. Does longer time mean a better improvement of semen quality, or meaning waiving of varicocelectomy? For managing infertility related to varicocele, it is critical to investigate the best waiting time for better semen quality improvement. Hence, we performed this meta-analysis to find out the period when semen quality is optimal after varicocelectomy and to provide statistically significant data to guide patients on the timing of conception.

## Materials and methods

We performed the present meta-analysis in accordance with the preferred reporting items of the systematic review and meta-analysis protocols (PRISMA-P) [22]. There was no need to obtain ethical approval and informed consent, owing to the nature of the meta-analysis. The protocol of our systematic review and meta-analysis was registered in the International Prospective Register of Systematic Reviews (CRD42021249994).

### Search strategy

An extensive search was conducted in PubMed, Web of Science, and Cochrane Library from the inception dates to 31 March 2024, to identify studies regarding the varicocelectomy on semen parameters over time after surgery. The keywords used for our search were (“varicocele” OR “varicocelectomy” OR “varicocele repair”) AND (“semen parameters” OR “semen quality” OR “semen” OR “sperm” OR “sperm parameters” OR “sperm quality”) AND (“time” OR “months”). The search strategy was adapted to the requests of different electronic databases. The references list of the retrieved research was manually searched to find more eligible studies. The date of the last search was set at March 31, 2024, with no language restrictions applied. First, an initial screening was conducted independently by two writers (Y Mei and R Xu) based on the titles and abstracts. The ineligible studies were excluded for precise reasons. Secondly, the eligibility of all potentially related research was reviewed in full text and final confirmed during the data extraction process. Any disagreements were settled by consensus or in consultation with a third reviewer (X Feng).

### Inclusion and exclusion criteria

Studies included in our meta-analysis should satisfy all the following inclusion criteria: (a) the studies conducted in human beings and aged older than 18y; (b) the studies compared the semen quality in varicocele patients between pre-surgery and different durations after surgery; (c) the studies possessed sufficient data to pool results; (d) sperm parameters were assessed based on the World Health Organization (WHO) 2010 guideline. Studies were excluded if: (a) insufficient data or duplicated data; (b) patients aged younger than 18y; (c)study population undergoing surgery not for infertility; (d)review, meta-analysis, case reports, meeting abstracts, editorial comments, expert opinions, animal tests; (e) semen analysis unrelated to time after surgery. For studies published more than once, studies reporting the most informative and sufficient data were included in our meta-analysis.

### Data extraction and quality assessment

The following details were independently extracted by two writers (Y Mei and N Ji): name of the first author, publication year, and location of recruitment, design of the study, size of the sample, age of the population, follow-up duration, sperm paraments (sperm motility, semen volume, total motile sperm count, sperm concentration, sperm morphology).

The Newcastle–Ottawa Scale (NOS) tool [23] was used to evaluate the quality of included cohort studies by two authors (Y Mei and X Feng) independently. Selection, comparability, and outcome were the main parameters of the NOS tool, with a total score of 7 or more indicating a high-quality study, and studies with a score of less than 3 were considered low-quality studies. If the two authors draw inconsonant score, the study would be reevaluated by the third senior reviewer (D Xue).

### Statistical analysis

The statistical analysis and meta-analysis were conducted using STATA 16.0 (Stata Corp, College Station, TX, USA). The *P* value < 0.05 was considered to be statistically significant. Pre-surgery and post-surgery semen parameters were compared. The weighted mean difference (WMD) and 95% confidence interval (CI) were calculated to estimate the combined effect. The Z test and P value were used to measure the significance of the pooled results. The inconsistency (*I*^2^) statistics and Cochran *Q* test were used to assess the heterogeneity among the included studies. The fixed-effect model was used to pool data if there was no heterogeneity among studies (*P* > 0.05, or *I*^2^ < 50%), otherwise, the random-effect model was used. We performed sensitivity analyses, eliminating each individual study from the meta-analysis in each case to estimate the stability of the analyses and further analyses were performed in subgroups to explore sources of heterogeneity. We quantified the publication bias by Begg’s test. The publication bias existed if the P value of Begg’s test was < 0.05, on the contrary, it indicated no significant publication bias.

## Results

### Study selection results and basic characteristics

**Fig. 1:**
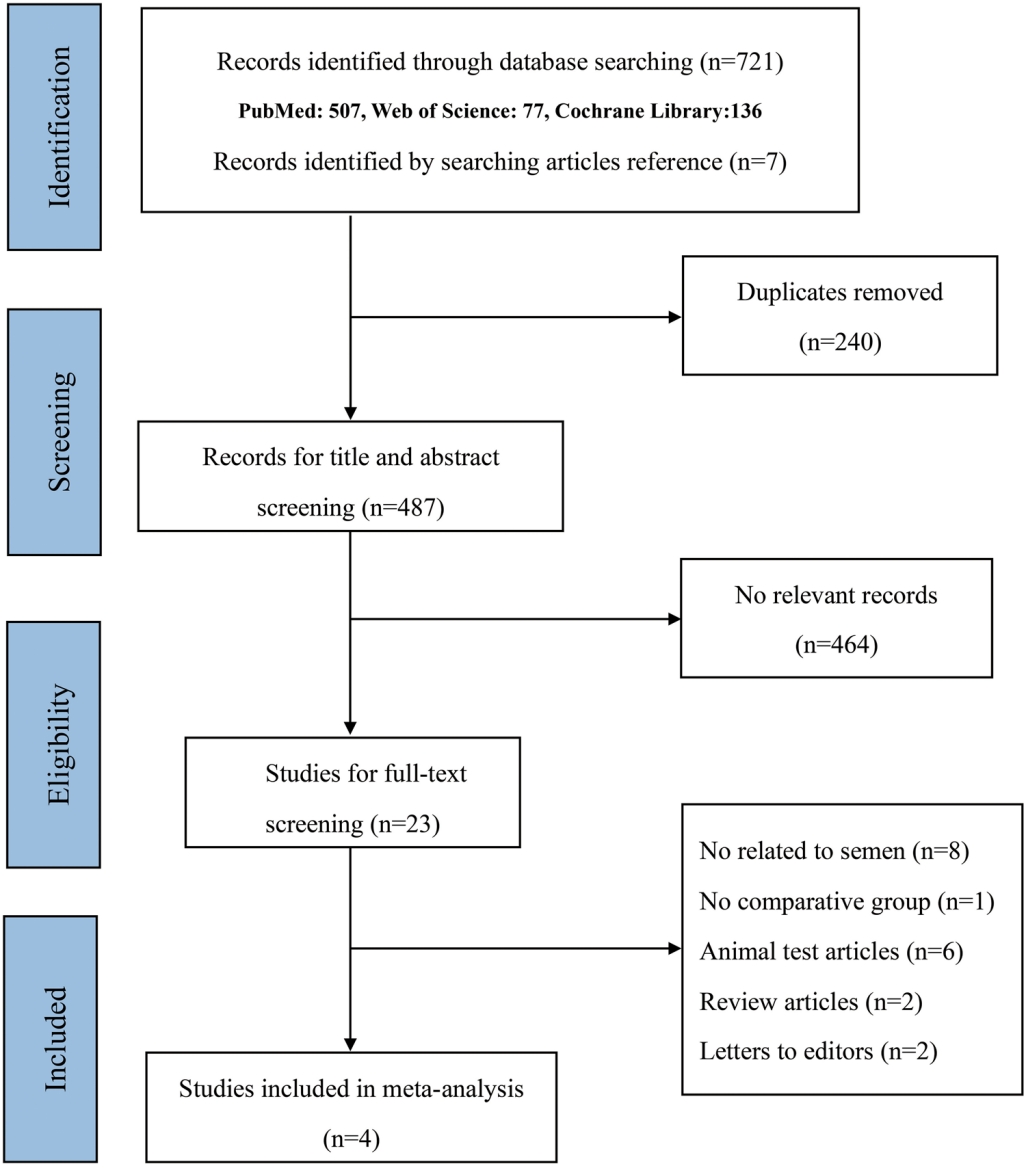
Flow diagram of the study selection process

We initially identified a total of 727 records related to our topic, including 507 records from PubMed, 137 records from Cochrane Library, 77 records from Web of Science. Six studies were identified by manually searching the reference lists of individual studies. First, we excluded 240 duplicated records by comparing title, authors, and publication year. Subsequently, a total of 464 records were excluded due to irrelevance, based on the content of their titles and abstracts. Then, 23 records remained for assessing the full-text eligibility. Upon reviewing the full texts of the remaining records, only four studies [2, 24–26] were included in our meta-analysis. Additionally, 19 articles were excluded for various reasons: eight were not related to semen (*N* = 8), one lacked a comparative group (*N* = 1), six were animal test articles (*N* = 6), two were review articles (*N* = 2), and two were letters to the editors (*N* = 2). The detailed study selection process is shown in Fig. 1. The publication year of the included studies ranged from 2012 to 2023, including a total of 279 varicocele patients. All these studies were prospective studies, and semen parameters were recorded pre-surgery, 3 months, 6 months, or 12 months after surgery. Table 1 detailed individual data regarding the name of the first author, year of publication, and location of recruitment, design of the study, size of the sample, age of the population, follow-up duration, and sperm parameters. The quality assessment results are shown in Table 2. All studies were regarded as high-quality studies based on NOS scores.

**Table 1:** Characteristics of studies included in the meta-analysis

First author (Year)	Country	Study design	Sample size	Age	Duration after varicocelectomy	Semen parameters
Bakri et al. (2012)[24]	Canada	Prospective study	100	36 ± 4.87	3 and 6 months	Semen volume
Fukuda et al. (2015) [2]	Japan	Prospective study	71	34.1 ± 5.3	3 and 12 months	Sperm concentration
Ghaed et al. (2020) [25]	Iran	Prospective study	100	29.5 ± 6.2	3 and 6 months	Sperm motility
Madhusoodanan et al. (2020) [26]	American	Prospective study	18	33.6 ± 8.9	3 and 12 months	Sperm morphology

**Table 2:** Quality assessment of studies included in the meta-analysis by using the Newcastle–Ottawa Scale

First author	Publication year	Selection	Comparability	Outcome	Total
Bakri et al. (2012) [24]	2012	4	2	2	8
Fukuda et al. (2015) [2]	2015	3	2	2	7
Ghaed et al. (2020) [25]	2020	3	2	2	7
Madhusoodanan et al. (2020) [26]	2020	3	2	2	7

### Sperm parameters in varicocele patients before surgery and 3 months after surgery

**Fig. 2:**
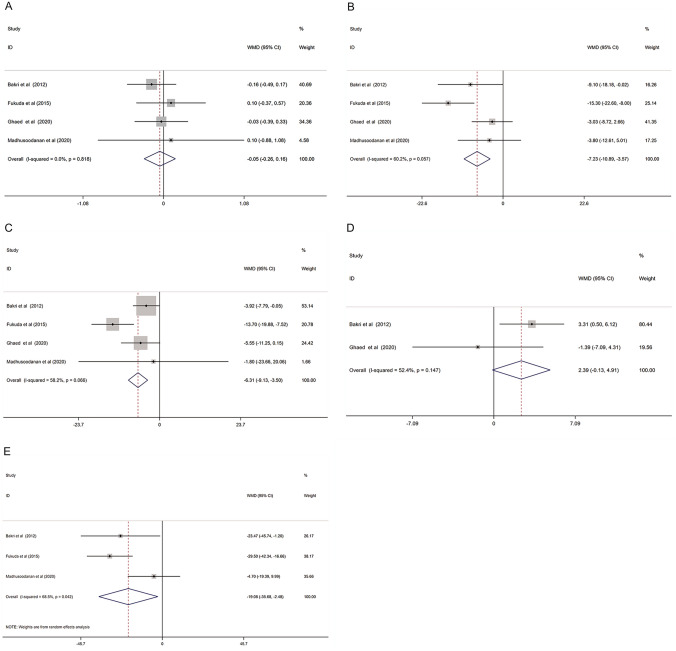
Forest plot and meta-analysis of semen parameters between pre-surgery and 3 months after varicocelectomy. (**A** Semen volume; **B** sperm concentration; **C**: sperm motility; **D** sperm morphology; **E** total motile sperm count)

All the semen parameters were compared between pre-surgery and 3 months after surgery. The results were pooled to calculate the WMD and 95% CI. Random effects models or fixed effects models were used according to the heterogeneity among the included studies. The results were showed in Fig. 2.

#### Semen volume

The semen volumes were reported in all included studies. The fixed-effect model was used to pool the data, based on the heterogeneity detection (*I*^2^ = 0.0%, *P* = 0.818). After varicocelectomy, no significant difference in semen volume was found between pre-surgery and 3 months after surgery (WMD: − 0.05 (− 0.26, 0.16)) (Fig. 2A).

#### Sperm concentration

Similarly, all eligible studies reported the sperm concentration. We still compared the sperm concentration between pre-surgery and 3 months after surgery. It increased by 7.23 million per ML when 3 months after varicocelectomy (WMD: − 7.23 (− 10.89, − 3.57)). Moderate heterogeneity was detected among studies (I^2^ = 60.7%, *P* = 0.057) (Fig. 2B).

#### Sperm motility

Sperm motility was compared between pre-surgery and 3 months after varicocelectomy. The results showed that sperm motility increased by 6.31% when 3 months after varicocelectomy (WMD: − 6.31 (− 9.13, − 3.50)), with medium heterogeneity (I^2^ = 58.2%, *P* = 0.066) (Fig. 2C).

#### Sperm morphology

Only 2 out of 4 studies reported sperm morphology between pre-surgery and 3 months after varicocelectomy. However, no significant improvement in sperm morphology was revealed by meta-analysis (WMD: 2.39 (− 0.13, 4.91)). And no significant heterogeneity was detected among the 2 studies (I^2^ = 52.4%, *P* = 0.147) (Fig. 2D).

#### Sperm total motile count

Three studies reported the total motile sperm count (TMSC). The results showed a significant increase of TMSC when detecting in 3 months after surgery (WMD: − 19.08 (− 35.68, − 2.48)), but the degree of heterogeneity was high (*I*^2^ = 68.6%, *P* = 0.042) (Fig. 2E).

### Sperm parameters in varicocele patients before surgery and 6 or 12 months after surgery

**Fig. 3:**
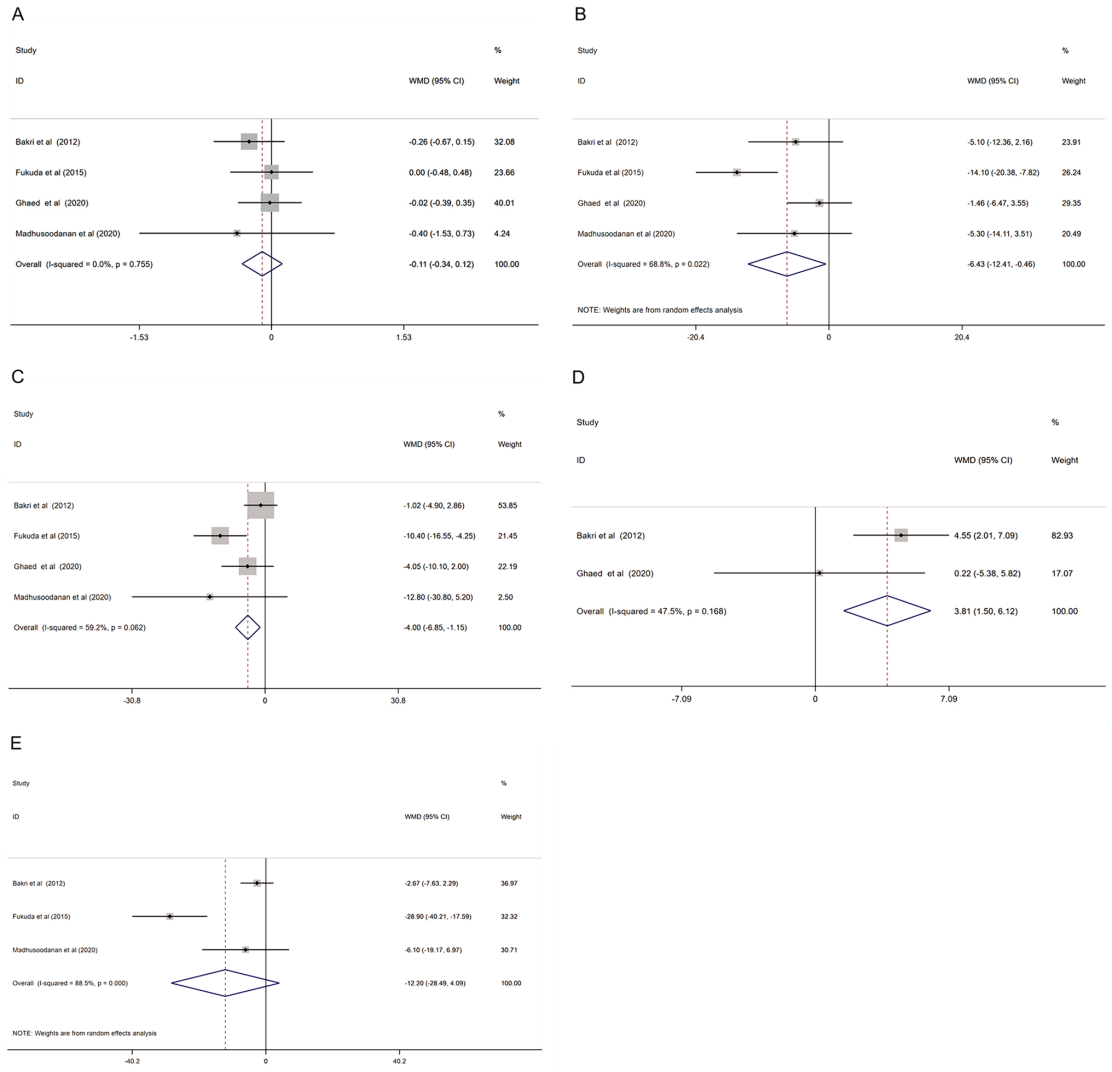
Forest plot and meta-analysis of semen parameters between pre-surgery and longer time (6 or 12 months) after varicocelectomy. (**A** Semen volume; **B** sperm concentration; **C** sperm motility; **D** sperm morphology; **E** total motile sperm count)

Similar semen parameters were compared between pre-surgery and 6 or 12 months after surgery. The pooled results are shown in Fig. 3. All the results showed that semen parameters improvements of 6 or 12 months after surgery were lower than those of 3 months after surgery.

#### Semen volume

Four studies reported the semen volume in varicocele patients between pre-surgery and 6 or 12 months after surgery. The pooled results showed no significant improvement in semen volume with a WMD of − 0.11 (− 0.34, 0.12), and the degree of heterogeneity was acceptable (I^2^ = 0.0%, *P* = 0.755). (Fig. 3A).

#### Sperm concentration

Four studies provided data about sperm concentration. The meta-analysis result of the random-effect model indicated that the sperm concentration of 6 or 12 months after surgery in varicocele patients was higher than that before surgery (WMD: − 6.43 (− 12.41, − 0.46). The heterogeneity was significant based on *I*^2^ value (*I*^2^ = 68.8%, *P* = 0.022) (Fig. 3B).

#### Sperm motility

Four studies reported sperm motility before surgery and 6 or 12 months after surgery. The results showed that sperm motility increased by 4.00% when 6 or 12 months after varicocelectomy (WMD: − 4.00 (− 6.85, − 1.15)), with medium heterogeneity (*I*^2^ = 59.2%, *P* = 0.062) (Fig. 3C).

#### Sperm morphology

Only 2 out of 4 studies reported the sperm morphology between pre-surgery and 6 or 12 months after varicocelectomy. However, the sperm morphology of 6 or 12 months after surgery showed a significant decrease when compared to sperm morphology before surgery (WMD: 3.81 (1.50, 6.12)), and the heterogeneity was acceptable (*I*^2^ = 47.5%, *P* = 0.168) (Fig. 3D).

#### Sperm total motile count

Three studies reported the total motile sperm count. The results showed no significant increase of TMSC when detecting in 6 or 12 months after surgery (WMD: − 12.20(− 28.49, 4.09)), but the degree of heterogeneity was high (*I*^2^ = 68.6%, *P* = 0.042) (Fig. 3E).

### Sperm parameters in varicocele patients 3 months after surgery and 6 or 12 months after surgery

**Fig. 4:**
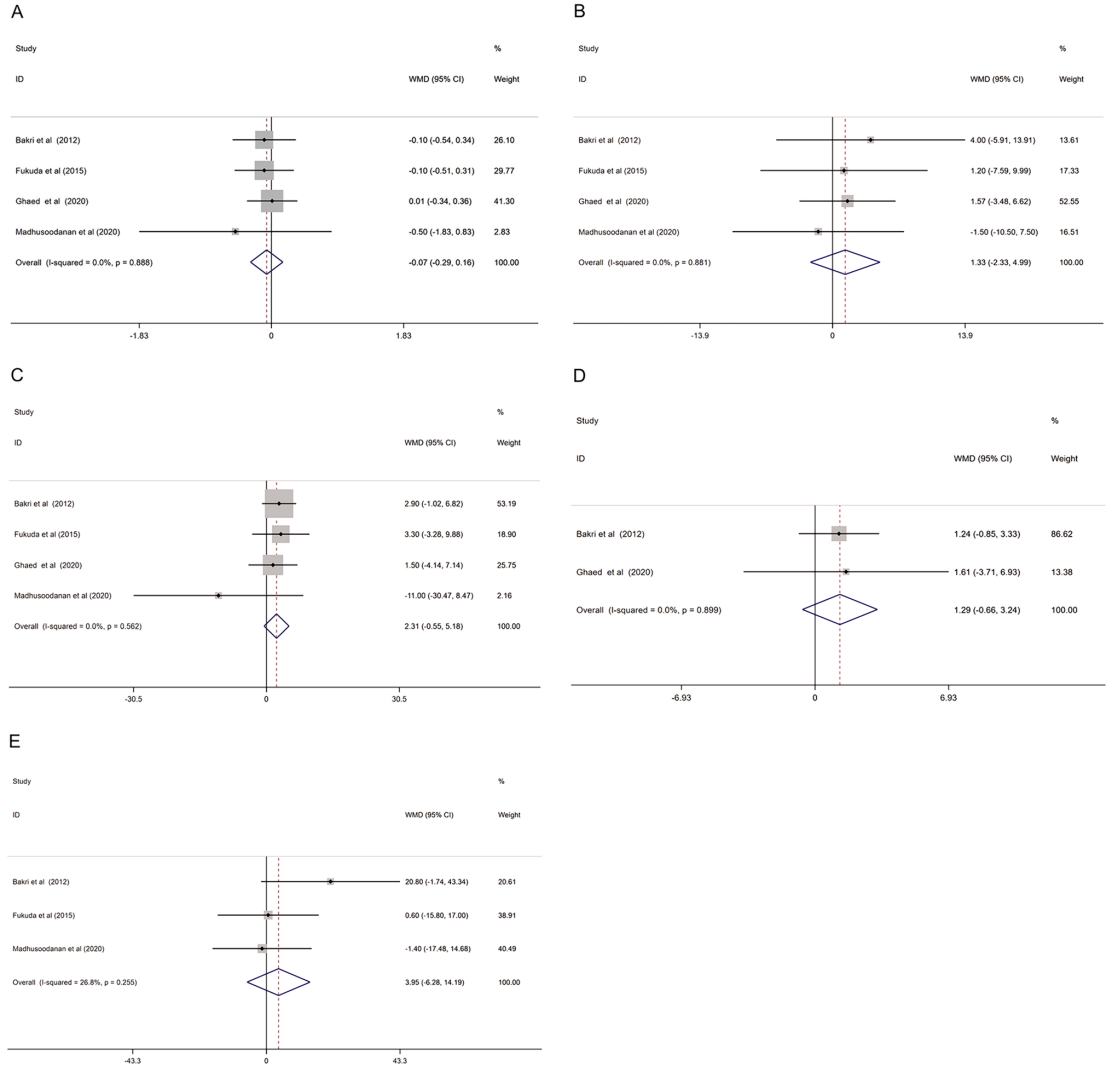
Forest plot and meta-analysis of semen parameters between 3 months after varicocelectomy and longer time (6 or 12 months) after varicocelectomy. (**A** Semen volume; **B** sperm concentration; **C** sperm motility; **D** sperm morphology; **E** total motile sperm count)

All eligible studies reported the semen parameters of varicocele patients between 3 months after surgery and 6 or 12 months after surgery. The meta-analysis results of these comparisons are shown in Fig. 4.

#### Semen volume

Four studies reported the semen volume in varicocele patients between 3 months after surgery and 6 or 12 months after surgery. The pooled results showed no significant difference in semen volume with a WMD of − 0.07 (− 0.29, 0.16), and the degree of heterogeneity was acceptable (*I*^2^ = 0.0%, *P* = 0.888) (Fig. 4A).

#### Sperm concentration

A total of four studies involved data on sperm concentration. The meta-analysis result of the fixed-effect model indicated that the sperm concentration of 6 or 12 months after surgery in varicocele patients was similar to those 3 months after surgery (WMD: − 1.33 (− 2.33, − 4.99). The heterogeneity was significantly based on the *I*^2^ value (*I*^2^ = 0.0%, *P* = 0.881) (Fig. 4B).

#### Sperm motility

Four studies reported sperm motility between 3 months after surgery and 6 or 12 months after surgery. The results showed that sperm motility had no significant difference between the two times (WMD: 2.31 (− 0.55, 5.18)), with negative heterogeneity (*I*^2^ = 0.0%, *P* = 0.562) (Fig. 4C).

#### Sperm morphology

Two studies reported sperm morphology between 3 months after surgery and 6 or 12 months after varicocelectomy. However, the sperm morphology of 6 or 12 months after surgery showed no significant difference when compared to sperm morphology 3 months after surgery (WMD: 1.29 (− 0.66, 3.24)), and the heterogeneity was acceptable (*I*^2^ = 0.0%, *P* = 0.899) (Fig. 4D).

#### Sperm total motile count

Three studies reported the total motile sperm count. The results showed no significant difference in TMSC when comparing TMSC between 3 months after surgery and 6 or 12 months after surgery (WMD: 3.95 (− 6.28, 14.19)), and the degree of heterogeneity was acceptable (*I*^2^ = 26.8%, *P* = 0.255) (Fig. 4E).

### Subgroup analysis of sperm parameters in varicocele patients 3 months after surgery and 6 or 12 months after varicocelectomy

**Fig. 5:**
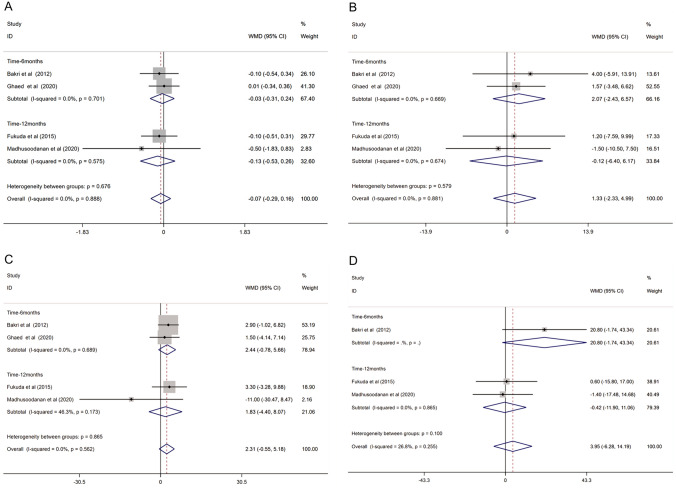
Subgroup analysis of semen parameters between 3 months after varicocelectomy and longer time (6 or 12 months) after varicocelectomy based on duration after surgery. (**A** Semen volume; **B** sperm concentration; **C** sperm motility; **D** total motile sperm count)

The subgroup analyses were performed only on the semen parameters between 3 months after surgery and longer time after surgery, which we were most concerned about. Notably, the degree of heterogeneity was acceptable. Time was the major factor that would influence the semen improvement. So, we conducted the subgroup analysis based on different months after surgery. Limited to the data on sperm morphology, no subgroup analysis was conducted to it. As shown in Fig. 5, all the semen parameters remained unchanged after subgroup analysis based on different times after surgery. Notably, the subgroup analysis revealed that there were no significant changes in semen parameters (semen volume, sperm concentration, sperm motility, and total motile sperm count) when comparing results 3 months after surgery to those at 6 months or 12 months after surgery.

### Sensitivity analysis and publication bias

**Fig. 6:**
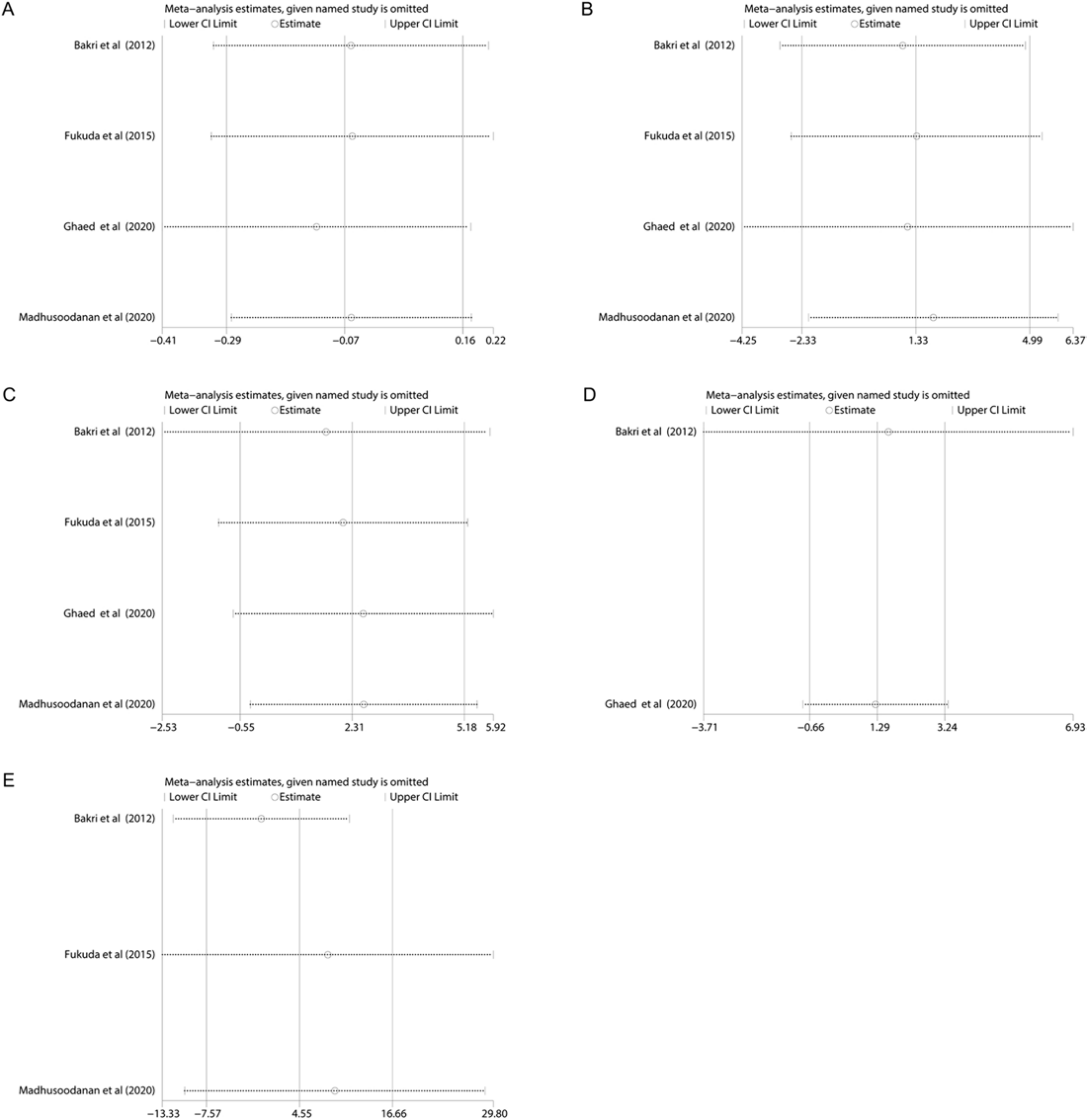
Sensitivity analysis of semen parameters comparison between 3 months after varicocelectomy and longer time (6 or 12 months) after varicocelectomy. (**A** Semen volume; **B** sperm concentration; **C** sperm motility; **D** sperm morphology; **E** total motile sperm count)

**Fig. 7:**
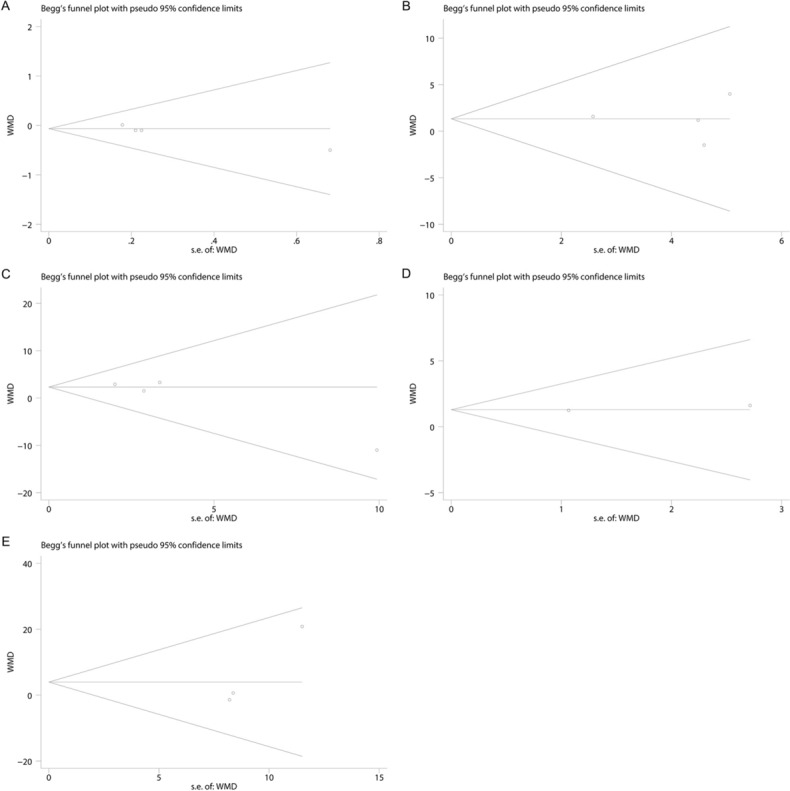
Publication bias of parameters comparison between 3 months after varicocelectomy and longer time (6 or 12 months) after varicocelectomy. (**A** Semen volume; **B** sperm concentration; **C** sperm motility; **D** sperm morphology; **E** total motile sperm count)

Similar to subgroup analysis, the sensitivity analysis and publication bias were conducted on the semen parameters comparison between 3 months after surgery and 6 or 12 months after surgery. As for sensitivity analysis, it was conducted by omitting one study in turn to detect the stability of pooled results and assess the influence of each study on the pooled results. The results were shown in Fig. 6, and indicated stable results of our meta-analysis. In addition, we found no significant publication bias by the Begg’s test, and the visual results are presented in Fig. 7.

## Discussion

Presently, the dominant opinion related the clinical varicocele to infertility tightly, based on the crude epidemiological findings of varicocele in the infertility population [27]. Studies conducted over a century concluded that the varicocele was related to inferior quality of semen, damaged sperm function, and testicular histology. No precise theory could completely explain the relationship between infertility and varicocele. Among several proposed mechanisms, the most commonly accepted one was that oxidative stress due to varicocele reduces spermatogenesis [28]. In addition, heat stress could also induce the production of reactive oxygen species, increasing the level of oxidative stress [29]. The oxidative stress could harm germ cells, influence non-spermatogenic cells, and the basal lamina of seminiferous tubules, ultimately resulting in changes in semen quality, including semen concentration, sperm morphology, and sperm motility [30].

Similar to the controversial literature related to infertility and varicocele, the relationship between varicocelectomy and improvement in semen quality remains controversial. However, the majority of published literature acknowledged the positive impact that varicocelectomy can achieve on semen quality, including original researches [31] and meta-analysis [13, 32, 33]. A Baazeem et al. [34] conducted a well-designed meta-analysis that included 22 studies to clarify the positive impact of varicocelectomy on semen quality. They concluded that varicocelectomy can improve sperm concentration (elevated by 12.32 × 106 sperm/mL, *P* < 0.0001), total motility (elevated by 10.86%, *P* < 0.0001), and progressive sperm motility (elevated by 9.69%, *P* = 0.003). Furthermore, several studies investigated whether varicocelectomy could improve the outcome of pregnancy, which is the most critically significant question [35–38]. It has been reported that there is an increase in spontaneous pregnancy rates after varicocelectomy [39]. Generally, the present evidence supports the favorable impact of varicocelectomy on the outcome of pregnancy.

However, fewer studies were conducted to explore the length of time after varicocele repair, expecting a better improvement of semen parameters. In 2012, Ayman et al. conducted the first prospective study to compare the semen parameters after varicocelectomy, based on different durations after surgery [24] They found that the semen quality would improve after varicocelectomy no matter at 3, 6, or 9 months. However, the semen parameters didn’t improve further by 6 months or longer. Then, another study conducted by T. Fukuda et al. also compared the semen parameters at 3 and 12 months after varicocele repair. The results were consistent with the former one. The two prospective studies concluded that the initial improvement of semen parameters after varicocelectomy should occur at least 3 months after surgery. However, no further improvement will be accomplished when waiting for more than 3 months. Our meta-analysis enrolled more varicocele population showed that semen parameters before surgery were inferior to that at 3, 6, 12, and longer time after surgery. It still validates the positive impact on semen parameters of varicocele patients by varicocelectomy. However, when comparing the semen parameters among different durations after surgery, we found that no further improvements were found based on longer waiting time after surgery. The improvement of semen quality after surgery should be time-dependent.

Considering that time is the key factor influencing the improvement degree of semen quality, we conducted a subgroup analysis based on the different durations after varicocele repair. When comparing the semen quality between 3 months and longer time after surgery, the semen quality didn’t show any further improvement in longer time after surgery. Actually, another prospective study [25] was conducted to compare semen parameters between 3 and 6 months after surgery, regarding semen volume, sperm concentration, sperm morphology, and progressive motile sperm count. No statistical differences in these parameters were found between the two different durations after surgery. Although Vinayak et al. [26] considered that the semen parameters improvement would continue for more than 12 months. They just compared the semen paraments between baseline and 12 months. The limitation of their study is the lack of comparison of semen parameters for different durations after surgery.

Previous studies just verified that varicocelectomy would potentially improve the semen quality and spontaneous pregnancy outcomes of clinical varicocele patients. With the development of assistant reproductive technology (ART), several methods have been approved for men with clinical varicocele combined with infertility, which includes in vitro fertilization (IVF), intrauterine insemination (IUI), or intracytoplasmic sperm injection (ICSI) [40]. Moreover, it has been proved that these options could improve the chance of conceiving for infertility couples [41]. So, for elderly couples, ART would be superior to varicocelectomy based on efficacy. However, they would miss the benefit of varicocele, when they turned to ART. Accordingly, it is critical for couples to find the best time to wait for better improvement of semen parameters.

Our results supply a best waiting time after varicocele surgery for patients and clinicians. For couples with varicocele-related infertility, varicocelectomy should be supplied to the men. And they could turn to ART when no obvious improvements in semen parameters were observed 3 months after surgery.

Limited to available data, no further analyses were conducted to explore the basic characteristics of semen quality on the best time waiting for the improvement. But, Teruo et al. conducted it based on the baseline TMSC.2 They found that the time-dependent changes of semen parameters showed similar results based on different baseline TMSC (< 3 million, 3–9 million, > 9 million respectively). On the contrary, another prospective study [42] demonstrated that the improvement of semen quality would continue up to 12 months after surgery based on the baseline TMSC > 9 million, compared to the baseline TMSC < 5 million or ranging 5 million to 9 million. The controversial conclusion means that further additional well-designed research is needed to evaluate the effect of baseline semen characteristics on the time-dependent changes of semen parameters after surgery.

To our knowledge, the present meta-analysis is the first one to explore the effect of time on semen parameters after varicocelectomy. The former meta-analyses mainly concentrated on the following comparisons: [1] compared the semen parameters before and after varicocelectomy [13]; [2] compared semen parameters between bilateral varicocelectomy and unilateral varicocelectomy [43]; [3] compared the semen parameters among different operation methods [44]. Less importance was attached to time after varicocelectomy. Our results would greatly remind couples and physicians to pay attention to the best time for semen parameters improvement, and better managing infertility after varicocelectomy. However, several limitations should be mentioned when interpreting our results. First, the number of studies included in our meta-analysis was small, which limited statistical power. Second, not all studies included all sperm parameters (semen volume, sperm motility, sperm concentration, and sperm morphology). However, all eligible studies for this meta-analysis were considered to be high-quality studies.

## Conclusions

The findings of our meta-analysis showed that the semen parameters following varicocelectomy would be maintained for 3 months, 6 months, and 12 months. However, semen parameters do not show further improvement beyond three months post-varicocelectomy when compared to results observed at six months or later. Consequently, three months following varicocelectomy may be the best time to conceive for managing varicocele-related infertility. It also alerted the physicians that time is essential to varicocele patients after varicocelectomy. Notably, more well-designed prospective studies are needed in the future to validate our conclusion.

## Data Availability

Data can be obtained from the corresponding author upon reasonable request.
